# Assessment of Mediterranean Diet Adherence and Lifestyle Change during COVID-19 National Lockdown in Tunisian Adult Population

**DOI:** 10.3390/nu14194151

**Published:** 2022-10-06

**Authors:** Saoussen Turki, Khaoula Bouzekri, Tarek Trabelsi, Jalila El Ati

**Affiliations:** 1 SURVEN (Nutrition Surveillance and Epidemiology in Tunisia) Research Laboratory, 11 Rue Jebel Lakhdar, Bab Saadoun, Tunis 1007, Tunisia; 2 High Institute of Medical Technologies, University Tunis El Manar, 9, Rue Docteur Zouheïr Safi, Tunis 1006, Tunisia; 3 INNTA (National Institute of Nutrition and Food Technology), 11 Rue Jebel Lakhdar, Bab Saadoun, Tunis 1007, Tunisia

**Keywords:** COVID-19, lockdown, MD adherence, lifestyle change, Tunisian adults

## Abstract

The Mediterranean diet (MD) is a plant-based diet associated with a reduction in the risk of developing COVID-19 comorbidities. Lockdown instigation during the COVID-19 pandemic has affected eating habits and lifestyles, highlighting the need to analyze the healthiness of new consumption patterns. We conducted a survey to assess lifestyle change in Tunisian adults and their MD adherence. A total of 1082 respondents completed a self-administered online survey designed to assess their food and lifestyle habits. Poor overall adherence to MD was observed (mean MEDAS score 6.6, SD 1.07) in a preponderance of the mid-MD adherent subgroup (71.2% of the participants). Location, age, profession, and household welfare proxy were the main determinants of high MD adherence. When adjusting for sociodemographic variables, location and income remained statistically significant. Positive health outcomes were noticed in respondents with high MEDAS scores. Most importantly, binary logistic regression showed that risk of COVID-19 infection decreased as MEDAS score increased for unvaccinated obese participants (OR = 0.63; confidence interval (CI) 0.4–0.98; *p* = 0.045). Regarding lifestyle changes, confinement had contributed to an overall reduction in cigarette consumption, sleeping hours, and physical activity. Long-term consequences of these changes on health outcomes must be further explored.

## 1. Introduction

In December 2019, a new infectious respiratory disease was first reported in Wuhan, Hubei Province, China. Caused by a new class of virus (SARS-CoV-2), it was recognized by the World Health Organization (WHO) as COVID-19 [1]. At the time of writing, this paper (3 August 2022), more than 575 million confirmed cases and 6 million deaths have been reported worldwide [2]. Globally, Tunisia experienced five waves of coronavirus. Breaking a record number of daily contaminations, the country was ranked on 6 July 2021 as the first in the Arab world and Africa in terms of number of contaminations and deaths [3]. To mitigate the spread of the disease, a variety of measures have been implemented, such as the banning of all social activities and cancellation of public events, up to the establishment of curfews and partial and complete lockdowns. These restrictive measures have influenced people’s lifestyle and dietary habits [4]. In a recent cross-sectional study, we showed that of 1082 Tunisian adults, 57.8% reported a change in their eating habits [5].

In the territories around the Mediterranean basin, “Mediterranean diet” (MD) is a term that describes the traditional eating habits of people living in olive-growing areas [5]. UNESCO recognized the MD as a Cultural Heritage of Humanity in 2010 [6,7].

The MD is commonly accepted as a likely dietary model for the prevention and control of chronic noncommunicable diseases throughout life, and the protective effect of the MD against these diseases has been accurately reported by many studies [8,9,10,11]. The association between adherence to the Mediterranean diet and physical health function, controlling for confounding effects of age, smoking, BMI, alcohol consumption, and education, has been suggested to contribute to its beneficial effects [12]. In a recent ecological study, a negative association between MD adherence and COVID-19 cases and related deaths was documented in Spain and across 23 OECD (Organization of Economic Co-operation and Development) countries [13].

Despite its promotional health effects, the literature suggests that adherence to the MD has declined in the last few years in most regions of the world [14,15]. Globalization and cultural and social changes have caused a progressive abandonment of the MD and a simultaneous shift towards the Western diet [15]. This trend may have reversed or at least slowed during the initial phases of the current pandemic, as described in a recent systematic review of 12 observational studies [16].

Since the MD is generally associated with overall well-being and MD adherence is associated with reduction in risk of common comorbidities observed in COVID-19 patients, the aim of this study was to assess adherence to the MD among a large sample of the general Tunisian adult population along with their lifestyle changes, namely, physical activity, smoking status, and sleeping hours, during the COVID-19 lockdown period.

## 2. Subjects and Methods

### 2.1. Selection of Participants and Study Design

The study was carried out by the SURVEN research team (Nutrition Surveillance and Epidemiology in Tunisia) from the National Institute of Nutrition and Food Technology in Tunisia. It was a cross-sectional study based on a self-administered questionnaire with a nonprobabilistic sampling method. A Google Forms questionnaire was disseminated to the Tunisian population aged between 20 and 74 years through social networks (Facebook and Instagram) as well as institutional and private mailing lists. To calculate the sample size, the data published by the INS in 2021 were taken as reference. The Tunisian population was estimated at 12,075,950 million inhabitants [17]. Accordingly, the calculation of the sample size was carried out with 95% confidence level and 3% precision, and since the expected proportion of the change in population was unknown, 0.5 proportion was selected. The theoretical sample size was 1067 subjects [18]. Participation in the study was completely free, voluntary, and anonymous with the informed consent of the participants on data sharing and confidentiality policy. No personal data were requested, in accordance with the laws on the protection of personal data and the guarantee of digital rights. Therefore, this online survey did not require ethics committee approval.

### 2.2. Questionnaire Design

The questionnaire was established using Google Forms and disseminated in French and Arabic languages to meet the acceptance and understanding of the Tunisian population. The first section was dedicated to sociodemographic and economic data collection, and the second section included anthropometric and medical data. Weight and height were reported by the participants and used for the calculation of the body mass index (BMI), expressed as kg/m^2^. For BMI below 18.5, the participant was considered underweight. For BMI between 18.5 and 24.9 the subject was classified as normal weight. For BMI between 25 and 29.9 the subject was considered overweight, and if BMI exceeded 30.0, the subject was considered obese. Data describing the general health status were recorded as well. Subjects suffering from multiple chronic diseases (more than 3 disease types) were considered of morbid health status.

Adherence to MD was assessed in the third section using the 14-item MEDAS screener [19]. According to the MEDAS screener responses, a MEDAS score was calculated, which ranges from 0 to 14. Based on this score, respondents were divided into three classes: (1) low MD adherence for scores ranging from 0 to 5, (2) medium MD adherence for scores from 6 to 9, and (3) high MD adherence for scores ≥10.

A fourth section included a structured questionnaire packet of 6 questions about daily consumption of certain foods not included in the MEDAS screener but important for MD pattern. The final section comprised 7 questions on lifestyle habits regarding smoking and sleeping habits, as well as physical activity. The full online version of the questionnaire is available in Appendix A. A pilot study on 20 women and 20 men of all age categories was launched and feedback collected to assess questionnaire quality.

### 2.3. Data Collection

Participants completed the forms directly connected to the Google platform. Once completed, each response was transmitted to this platform and the final database was downloaded as a Microsoft Excel spreadsheet. The questionnaire was disseminated over two months (between 17 May and 20 July 2021), coinciding with the fourth wave of COVID-19 in Tunisia. No general lockdown measure had been instigated during that period, but intercity movements were prohibited, and a curfew was imposed from 10 p.m. to 5 a.m.

### 2.4. Statistical Analyses

Descriptive results are expressed as proportions (%) for categorical variables and means with standard deviation and medians with interquartile range between square brackets [IQR] for continuous variables. Shapiro–Wilk and skewness–kurtosis tests were performed to assess variable distribution. Chi-squared and Fisher exact tests were used to evaluate equality distributions between groups. Mann–Whitney U and Kruskal–Wallis tests were applied to compare continuous variables among two or more groups whenever normality was not confirmed. Whenever relevant, post hoc tests were applied for more accurate analyses. Spearman’s correlation coefficient was calculated for associated continuous variables. McNemar tests were performed to compare between categorical variables (smoking status, sleeping habits, and sports practice) before and during COVID-19. Finally, associations between independent variable coded as 2 category response variables (virus infection or exposure) and sociodemographic variables (age, sex, region, education, professional activity, household welfare proxy) were assessed using binary logistic regression models (odds ratio, OR). For independent variables coded as more than 2 category response variables (MD adherence) we used multinomial logistic regression models (relative risk ratio, RRR) to estimate the association with sociodemographic covariates. The type I error risk was set at 0.05 for all analyses. Statistical analyses were performed using Stata 14.0 (StataCorp, College Station, TX, USA) [20]. For statistical analyses, risk of COVID-19 infection, MEDAS score, smoking status, and sleeping habits were considered outcome variables, while sociodemographic factors, i.e., sex, age, location, education, occupational status, household size, household welfare proxy, and health status (BMI, incidence of chronic disease, COVID-19 infection, and MD adherence status) were considered exposure variables.

## 3. Results

### 3.1. Participant Characteristics

A total of 1121 participants completed the questionnaire and 1082 responses were validated, representing a response rate of 101%. Tunisian nationals aged between 20 and 74 years were included in the study. As shown in Table 1, the study sample covered all Tunisian regions. Women represented 74.3% of the study population and subjects aged between 25 and 60 years were the most prevalent group for both sexes. Other sociodemographic data are summarized in Table 1.

Anthropometrics of the population are reported in Table 2. Mean age was 32.5 ± 12.0 years. A normal BMI was found for 48.8% of the study population, 5.5% were classified as underweight, 31.1% as overweight, and 14.5% as obese, with no sex difference. With regard to general health status, 78.7% of the respondents declared not suffering from any chronic diseases. As shown in Table 2, endocrine, nutritional, or metabolic diseases (24.3%), hematological diseases (11.3%) and cardiovascular diseases (10.9%) were the most mentioned chronic diseases with no sex difference. The great majority of the respondents declared eating spontaneously and not following a diet.

With regard to COVID-19 incidence, 67.9% of the surveyed subjects confirmed not being infected by SARS-CoV-2 and 81.9% had not experienced a family death due to COVID-19. Only 21.7% had been vaccinated against the virus at the period of the survey.

Taking risk of COVID-19 infection as outcome variable and age, health status, sex and BMI as exposure variables, binary logistic regression showed that risk of COVID-19 infection significantly increased with the elderly group compared to young adults (OR = 5.8; confidence interval (CI) = 1.6–20.5; *p* = 0.006). Subjects with more chronic diseases also had a higher risk of infection than healthy subjects (OR = 1.49; CI = 1.07–2.07; *p* = 0.017). For our study sample, no relationship was found between COVID-19 infection and sex or BMI (*p* =0.71 and *p* = 0.83, respectively).

### 3.2. MD Adherence

After stratification of the study population in three classes based on calculated MEDAS score, MD adherence (outcome variable) was first analyzed according to sociodemographic factors (exposure variables). As shown in Table 3, the mean MEDAS score was 6.6 with standard deviation of 1.07 for the whole population with a preponderance of medium adherents (71.2%) compared to low adherents (23.7%) and high adherents (5.2%).

For the next analysis, we excluded subjects who reported following weight loss, therapeutic or vegetarian diets along with those who did not specify the diet. Hence, the sample size decreased to 806 individuals. The Mann–Whitney test showed no sex difference in MEDAS score (*p* = 0.24). An equal distribution of MD adherence classes among men and women was noticed as well. However, the Kruskal–Wallis test showed a significant difference in MEDAS score among geographical areas (*p* = 0.028), with the highest mean recorded in the center-east compared to Greater Tunis (post hoc analysis *p* = 0.021). It should also be stressed that the center-east presented the highest percentage (10.2%) of high MD adherents compared to other regions, whereas no high MD adherents were observed in the center-west, and a low percentage of this subpopulation was noticed in northwest Tunisia (1.5%).

**Figure 1:**
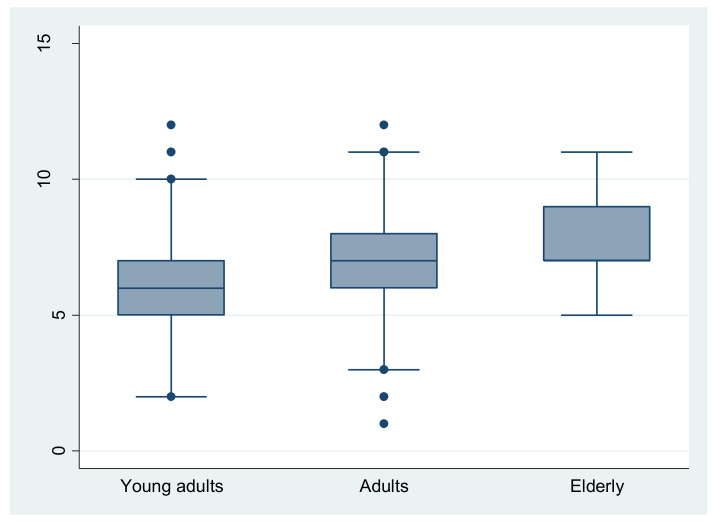
Distribution of MEDAS score over age categories. Elderly subjects had significantly higher MEDAS scores than younger groups (post hoc analysis *p* < 0.001, *p* < 0.001 for adults and young adults, respectively).

A significant difference (*p* < 0.001) in MEDAS score was found among different age-groups (Figure 1) with positive and significant Spearman correlation coefficients (r = 0.15, *p* < 0.001). Elderly subjects presented significantly higher MEDAS scores than younger groups (post hoc analysis *p* < 0.001, *p* < 0.001 for adults and young adults, respectively). In agreement with this result, the elderly group had a significantly (*p* < 0.001) higher proportion of high MD adherents (16.6%) than the other groups (3% and 5.1% for young adults and adults, respectively).

Unlike education, occupation made a significant difference in MEDAS score (*p* = 0.021). When post hoc analysis was performed, a significantly higher score for upper executives was found compared to students (*p* = 0.003) and middle executives (*p* = 0.05). Spearman’s test demonstrated a positive significant correlation between MEDAS score and employment (r = 0.15, *p* < 0.001).

**Figure 2:**
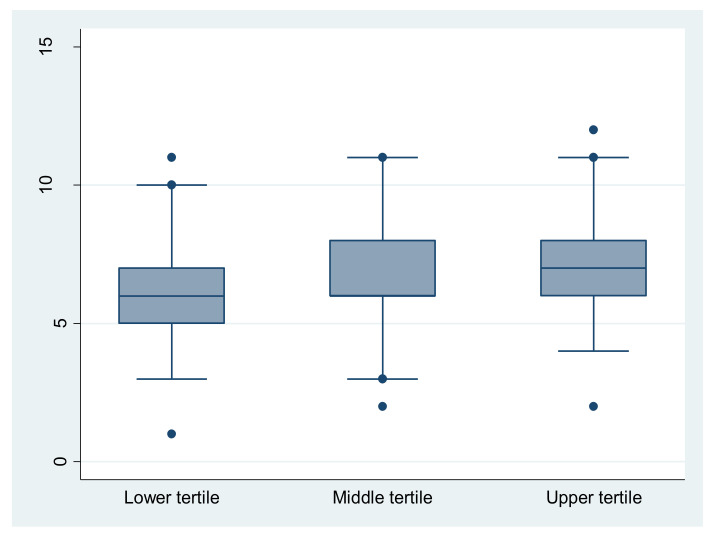
Distribution of MEDAS score over household welfare proxy. Significant difference for the mean MEDAS score between the three economic classes (*p* < 0.001). A post hoc analysis showed that a significantly higher mean score is noticed for high economic level (upper tertile) compared to medium (*p* = 0.025) and low economic classes (*p* < 0.001).

No significant difference (*p* = 0.2) between single, small, and large households was found for MEDAS score or distribution of MD adherents’ classes. By contrast, with regard to welfare proxy parameter, our results showed a significant difference for the mean MEDAS score between the three economic classes (*p* < 0.001). A post hoc analysis showed that a significantly higher mean score is noticed for high economic level (upper tertile) compared to medium (*p* = 0.025) and low economic classes (*p* < 0.001) (Figure 2). Spearman’s test confirmed the positive and statistically significant correlation between the participant’s economic level and their MEDAS score (r = 0.214, *p* <0.001). The proportion of high-MD adherents (7.3%) was significantly preponderant (*p* < 0.001) among high economic class compared to medium (4.6%) and low economic classes (3.4%).

Multivariate logistic regression revealed that compared to Greater Tunis, respondents living in central east Tunisia were more likely to be high MD adherent (RRR = 4.51; 95% IC: 1.54–13.2; *p*= 0.006) along with elderly participants (RRR = 49.8; 95% (IC): 4.7–522.8; *p* < 0.001). Participants in the upper tertile of household welfare proxy were predicted to be highly MD-adherent in comparison with the lower tertile (RRR = 5.32; 95% (IC): 1.66–17; *p* = 0.005). To minimize the effect of selection bias, a classified analysis based on sex, age, education, occupation, region, household size, and economic level was conducted. Results showed that after adjustment on these variables, MD adherence remained significantly impacted by location and household welfare proxy. Participants living in central Tunisia were prone to be highly MD-adherent compared to those living in Greater Tunis (OR = 1.75; 95% (IC): 1.16–2.65; *p* = 0.007). Compared to the lower tertile, participants in the middle tertile of household welfare proxy were predicted to be highly MD-adherent (OR = 1.96; 95% (IC): 1.38–2.79; *p* < 0.001) along with those in the upper tertile (OR = 2.64; 95% (IC): 1.76–3.96; *p* < 0.001).

**Figure 3:**
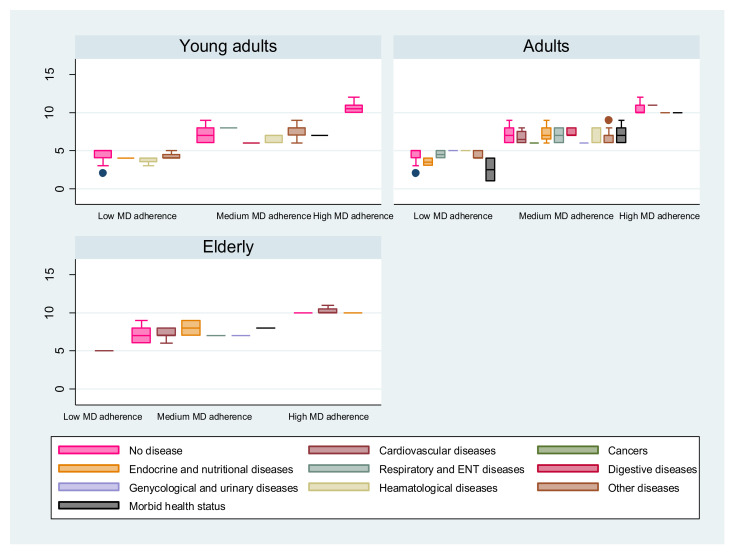
Impact of MD adherence on health status for the three MD-adherence profiles. Distribution of MEDAS score over MD adherence by age category and chronic diseases declared by participants. Kruskal–Wallis tests showed a significant difference in MEDAS score related to health status considered (*p* = 0.047). For all age categories, healthy participants recorded the highest MEDAS scores. Adults with morbid health status with multiple chronic diseases recorded the lowest MEDAS scores.

Kruskal–Wallis tests showed a significant difference in MEDAS score (outcome variable) related to health status considered as exposure variable (*p* = 0.047). As shown in Figure 3, the highest MEDAS score was recorded by healthy adults while the lowest score was observed among adults presenting a morbid health status with multiple chronic diseases. For the elderly group, cardiovascular, endocrine, nutritional and metabolic diseases were the only diseases reported by high MD adherents (Figure 3).

In agreement with those results, for adults and young adults, high MD adherents reported good health status with no relevant chronic diseases (unshown data). For elderly, high MD adherents reported less affection by chronic diseases than medium adherents. Cardiovascular and endocrine diseases were the only diseases declared by this group (Figure 3).

**Figure 4:**
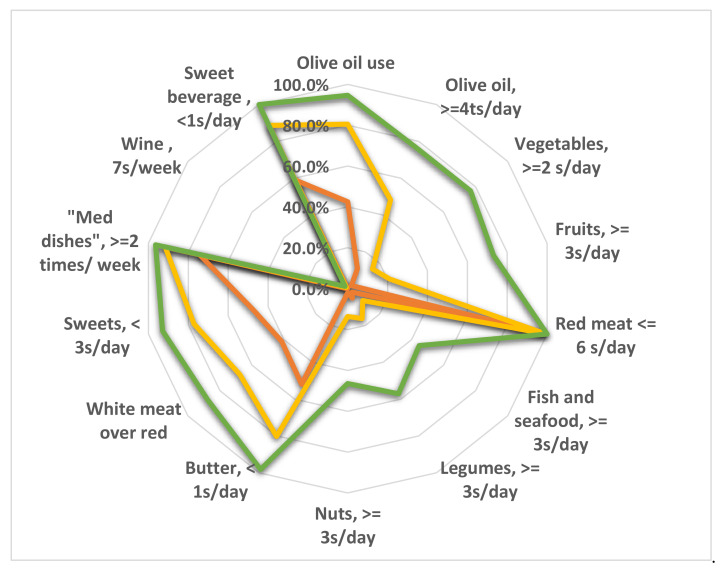
Compliance with items from PREDIMED questionnaire according to adherence profile: high (green), medium (yellow) and low adherence (red) to the Mediterranean diet (MD). The radar chart plots the values of each item of the questionnaire along a separate axis. Each axis refers to one of the 14 recommendations of PREDIMED questionnaire. Axis starts in the center of the chart (0% compliance) and ends at the outer ring (100% compliance). The values are the percentage of respondents compliant with each recommendation. The figure shows that consumption of fish and seafood, legumes, nuts and wine are commonly inappropriate for the three MD-adherence profiles.

An analysis of the degree of compliance to the MD with each recommendation of the PREDIMED questionnaire for the three categories of MD adherence was carried out. As shown in Figure 4, a drastic difference in compliance was observed for almost all food groups except frequency of weekly preparation of Mediterranean dishes, which was comparable between the three adherence profiles (Figure 4). In addition, no difference was observed for the consumption of red meat and wine. This latter remains unadopted in the Tunisian culinary culture.

It is worth noting that high MD adherents were mostly noncompliant with the recommendations for the consumption of fish and seafood, legumes and nuts, with compliance below 60% (Figure 4). Comparison was carried out between the three adherence profiles for the consumption of foods belonging to the MD but not included in the PREDIMED questionnaire. For the three adherence profiles, no statistically significant difference was found for the consumption of cereals, wholemeal bread, cracked wheat, cooked wheat and corn, nor for milk, yoghurt, cheese, dairy products, or eggs (unshown data). However, a statistically significant and positive correlation was noted between the amount of water consumed and the degree of adherence to the MD (r = 0.13, *p* < 0.001).

For analyses related to COVID-19 infection, apart from subjects who declared following a specific diet, vaccinated subjects as well as those who suspected infection with no COVID test confirmation were also excluded from the subsequent analysis. To investigate correlation between COVID-19 infection (outcome variable) and MEDAS score (exposure variable), binary logistic regression showed that particularly for unvaccinated obese subjects with no specific diet (n = 87), as MEDAS score increased, the risk of being infected by COVID-19 significantly decreased (OR = 0.63; CI: 0.4–0.98; *p* = 0.045). The risk of infection significantly increased for obese subjects aged above 60 years (OR = 26.75; CI: 1.4–510.08; *p* = 0.029).

### 3.3. Lifestyle Habits

Regarding lifestyle habits, our study focused on changes in smoking, sleeping habits and physical activity before and after the occurrence of the COVID-19 pandemic.

*Smoking:* Overall, the study population reduced cigarette consumption during the confinement periods. Indeed, the number of nonsmokers increased slightly from 81.4% before the confinement to 83.27% during the confinement. It should also be noted that subjects smoking fewer than five cigarettes per day decreased from 4.5% to 3.4% and those smoking more than 10 cigarettes per day decreased from 10.3% to 8.9% (Table 4). This was confirmed by the McNemar test (McNemar value = 2.64, *p* = 0.0018).

The Mann–Whitney test showed that sex (exposure variable) determines smoking status (outcome variable) in a statistically significant way before and after confinement (*p* < 0.001). Indeed, there were more nonsmoking women than men for both the pre- and post-COVID-19 periods. Changes in smoking habits had mostly affected men. Proportion of men consuming more than 10 cigarettes per day decreased from 27.6% to 21.9% during lockdown. Before the pandemic, the majority of nonsmokers were young adults (86.4%) and elderly (84.6%). During the containment periods, adults reduced their cigarette consumption, increasing the percentage of nonsmokers of this age category from 78.3% to 81.9%. Prior to the pandemic, a negative association (r = −0.14, *p* < 0.001) between cigarette consumption and instruction level had been ruled and remained unchanged after the pandemic (r = −0.13, *p* < 0.001). The highest percentage of nonsmokers was observed among housewives (92%). This percentage remained unchanged during the confinement periods. Post-COVID showed for all occupational classes, except students and retirees, a decrease in the percentage of heavy smokers. Finally, our results showed that those who live alone tend to consume more cigarettes before and during the confinement. The proportion of lone heavy smokers was 24.1% (before lockdown) and 20.7% (during lockdown) higher than those living in small (9.9% and 8.5%) and large (9% and 8.6%) households.

*Sleeping:* Our results showed that confinement contributed to an overall reduction in sleep hours (Table 4). Those who slept less than 7 h per night increased after the pandemic from 31.2% to 38.9%. On the other hand, those who slept between 7 and 9 h/night decreased from 52.9% to 46.1% in a similar way to those who sleep more than 9 h/night, who decreased from 15.8% to 14.9% (McNemar value = 1.9, *p* < 0.001).

During confinement, women seemed to decrease their sleep hours. Those who slept less than 7 h during lockdown reached 39.2%. This is comparable to that of men (38.1%) whose sleep habits did not seem to be affected by confinement (unshown data).

A significant negative association between sleeping hours and age was shown before (r = −0.14, *p* < 0.001) and during confinement (r = −0.10, *p* = 0.0006). It should be noted that unlike the elderly, confinement led youth and adults to reduce their sleep hours.

A statistically significant difference was demonstrated for sleep patterns related to occupational status before and during the pandemic (*p* = 0.0001). Before the pandemic, the highest numbers sleeping less than 7 h per night were retirees (53.8%) and housewives (40%), while the lowest were students (23.4%) and workers (23.9%). Those sleeping more than 9 h were more likely to be unemployed (28.4%) compared to upper executives (8%). During confinement, many housewives slept less than 7 h (46%) along with middle executives (52.2%). Unemployed people also kept the habit of sleeping more than 9 h/night (32.6%) versus little among upper executives (7.3%).

*Physical activity:* Obviously, the pandemic significantly decreased physical activity (McNemar value = 5.29, *p* < 0.0001). Those not engaged in physical activity increased from 46.7% prepandemic to 74.9% within the pandemic (Table 5). Physical activity seemed to be sex-associated (*p* < 0.001). Prior to the pandemic, sedentariness among women was 48.6% and for men 41%. This trend does not seem to change after the pandemic, with an increase in sedentariness for both sexes, more for women (76.4%) than men (70.5%).

Cited by 27.1% of the participants, walking was the most popular activity before the pandemic. However, during the pandemic, as shown in Table 5, the top cited physical activity was training without weights (10.3%).

## 4. Discussion

This descriptive cross-sectional study was carried out at a critical period, considered to be the fourth wave of COVID-19 in Tunisia. During that period, the country recorded the highest rate of positive cases and deaths in Africa and the government limited freedom of citizens’ travel and movement [3]. Thus, the survey was based on a self-administered questionnaire disseminated through social networks (Facebook and Instagram) as well as institutional and private mailing lists. A high response rate was obtained, and the sample population had an accepted distribution in terms of age stratification and territorial coverage over the Tunisian regions compared to internet-user distribution in Tunisia [21]. A prevalence of women (74.3%) and respondents with higher education (91.8%) was noticed. Similar trends were reported in many surveys [22,23,24,25], explained by the fact that women and university-educated respondents are more likely to manifest interest in food and health studies [25].

Changes in diet and physical activity consequent to the COVID-19 lockdown can lead to an increase in the prevalence of several chronic diseases, such as obesity and diabetes, which are considered risk factors for mortality in patients with COVID-19 [23]. Effect of restrictive measures on healthy eating was operationalized in this study as adherence to the Mediterranean diet using the MEDAS screener. The MEDAS questionnaire has been recently validated as an effective tool to assess MD adherence over different countries in the Mediterranean region [26].

Our results showed that location, age, occupational status, and household welfare proxy were the main sociodemographic determinants of high adherence to the MD. Adjustment of results to minimize effect of selection bias showed that respondents living in central Tunisia and those in the upper tertile of household welfare proxy were predicted to have higher MD adherence. It should be stressed that in Tunisia, employment and investment are afflicted by regional disparities, especially between the well-developed coastal northern and eastern areas and the poorer, marginalized interior southern and western side of the country. Indeed, the coastal regions (central Tunisia) account for 90% of overall employment and 85% of the businesses operating in all sectors [27]. Moreover, like many countries all over the world, COVID-19 has stressed all segments of food supply chains, from farm production, food processing, transport and logistics, to final demand [28]. For example, the supply of fruit was adversely affected by the confinement and social distancing measures, as fruit farming is labor-intensive. Restrictions on movement of fruit farm workers sharply reduced fruit supply to local markets [29]. For similar reasons, the supply of fish and seafood to local markets might be sharply reduced especially for populations that are geographically distant from the coast [30]. This would explain the noncompliance with the recommendations for the consumption of fish and seafood observed in our study among the three classes of MD adherents. In addition to the health crisis, a deterioration in the social situation has signaled worsening economic regional disparities. Assuming a 5% decline in GDP and based on the precrisis poverty rate, the number of poor was expected to increase by 36.2% in 2020 [31]. This would correspond to an increase in the poverty rate from 15.2% to 20.7% of the total population. According to a recent report disseminated by the National Institute of Statistics, the poorest households have more difficulties than the better-off, with 62% of those in the lower quintile reporting that they are unable to meet all or part of their expenditures (versus 32% for the upper quintile) [32]. In January 2022, food prices increased by 7.6% year on year [32]. This might classify certain foods or food groups typical of the Mediterranean diet, such as nuts, olive oil, legumes fruits, vegetables, fish and seafoods as luxuries unaffordable for households of the lower and medium tertiles. For example, olive oil was estimated to be four times less consumed by the population living below the poverty line [33]. The price of fruit and legumes has increased by 80% since 2010 [34].

According to our findings, mean adherence to the MD among adults in Tunisia during the pandemic was not adequate and was lower than a healthy score (few high adherents (5.2%), mean MEDAS score 6.6/14 < 9). Consumption of fish and seafood, legumes and nuts were particularly inadequate for the three MD-adherence classes and has consequently to be primarily considered for an overall improvement in MD adherence. As explained previously, low incomes due to the economic crisis in Tunisia might also be contributing to the low MD adherence, for high adherence requires relatively high expenses [35].

Host nutritional status has been accepted as a key factor in the outcome of a variety of infectious diseases [36]. The literature has recently found a negative association between MD adherence and COVID-19 cases and related deaths [13,37]. According to our results, this inverse association remained robust within the subgroup of unvaccinated obese subjects with no specific diet. Moreover, we observed that the highest MEDAS scores were recorded by healthy adults, while the lowest were among adults presenting a morbid health status with multiple chronic diseases. This result confirms the protective role of this diet against many chronic diseases, such as cardiovascular diseases (arterial hypertension, myocardial infarction, heart attack, etc.), hematological diseases (anemia), endocrine, nutritional, and metabolic diseases (diabetes, hypothyroidism, hyperthyroidism, etc.) and other diseases that are considered common comorbidities in COVID-19 infection [38,39,40]. Low-grade chronic inflammation underlies COVID-19 comorbidities [40]. It has been proposed that as a plant-based dietary pattern, the MD has anti-inflammatory properties inherent to the high polyphenol content sourced from fruits, vegetables and extra-virgin olive oil [13].

Besides diet and food habits and behaviors, lifestyle changes during COVID-19 lockdown have been the focus of some studies [22,23]. Our study showed a decrease in the number of heavy smokers of 1.4%, i.e., 2.8 times higher than that reported by an Italian survey [22]. This decrease could be explained by a fear of the risk of COVID-19 aggravation and the evolution of the disease towards the severe form observed in heavy smokers [41]. According to our study, tobacco addiction seems to be higher among adults (35–60 years old) and those with low education (primary and secondary level). This could be explained by the social role that smoking plays in these subgroups, which had ended by social isolation due to confinement, as well as attempts to abandon smoking associated with the fear of a risk of complication in case of coronavirus exposure [42]. Meanwhile, these results may contrast with other studies in which smokers who were most stressed during waves of COVID-19 showed either an increase or decrease in smoking, suggesting that for some smokers, monotony and social isolation may have stimulated smoking, while for others, concern about contracting COVID-19 and becoming seriously ill may have motivated them to quit smoking [42]. Indeed, the subgroup who spent the confinement in solitary mode showed a higher dependence on tobacco compared to those living with their families. This is most likely due to the effect of monotony and boredom associated with the lack of social interaction, as perceived reduced social contact and health fears are linked to poor well-being [41].

With regard to sleep habits, we noticed an overall reduction in sleep hours, especially among women and the elderly. On the other hand, only the unemployed seemed to record more than 9 sleeping hours during the confinement period. The sex difference in the sleep disorder could be explained by higher perception of stress as well as a higher level of anxiety observed more significantly for women than men [43]. An association between stress and poor sleep has been reported, so that the reduction in sleep hours supports the hypothesis of a complex interaction between stress, sleep disturbance, and mental health during COVID-19 confinement [44]. In addition, a preference for late sleep has been observed for youth along with a delayed sleep phase as a result of the use of electronic devices and the decrease in social life [45] as well as the time spent watching TV and surfing on social networks [46], which may impair their perception of time.

Unsurprisingly, a significant decrease in physical activity has been reported during confinement. Nearly 28.3% of our study population gave up sports. As described in another surveys [47,48,49], outdoor activities such as walking, swimming, soccer, basketball and tennis, were suspended to give way to other home-practiced indoor activities but at a low practice rate.

This study is the first to be conducted in our country and could be considered complementary to another study focusing on the impact of the pandemic on food habits and behaviors [5]. Meanwhile, one of the main limitations of an online survey is that self-reported data can be subject to bias and misreporting. In addition, the use of snowball sampling through social media implies that the sample cannot be considered representative of the general Tunisian population, which leads to selection bias. Indeed, online tools limit access to people who are not used to this technology, such as the elderly and the uneducated. The main strength of cross-sectional studies relies on the fact that they are relatively quick and inexpensive to conduct. We need to consider that the main limitations of this kind of study include inability to make a causal inference and susceptibility to sampling bias, as they often need to select a sample of subjects from a large and heterogeneous study population. Thus, we raise awareness of the need to deeply explore the long-term consequences of COVID-19 confinement on dietary and lifestyle habits on a sample more representative of the Tunisian population.

## Figures and Tables

**Table 1:** Sociodemographic characteristics of the participants.

	Total Population *n* (%) ^1^	Women *n* (%)	Men *n* (%)
	1082 (100.0)	804 (74.3)	278 (25.7)
**Location**	***p =* 0.192** ^2^		
Greater Tunis	482 (44.6)	350 (43.5)	132 (47.5)
Northeast	154 (14.2)	120 (14.9)	34 (12.2)
Northwest	93 (8.6)	72 (9.0)	21 (7.5)
Center-east	195 (18.0)	151 (18.8)	44 (15.8)
Center-west	44 (4.1)	31 (3.9)	13 (4.7)
Southeast	90 (8.3)	67 (8.3)	23 (8.3)
Southwest	24 (2.2)	13 (1.6)	11 (4.0)
**Age category**	***p <* 0.0001** ^2^		
Young adult (20–25 years)	339 (31.3)	277 (34.4)	62 (22.3)
Adults (25–60 years)	691 (63.9)	503 (62.6)	188 (67.6)
Elderly (60 years and over)	52 (4.8)	24 (2.9)	28 (10.1)
**Education**	***p <* 0.0001** ^2^		
Not graduated	4 (0.4)	1 (0.1)	3 (1.1)
Primary school graduation	9 (0.8)	6 (0.7)	3 (1.1)
Secondary school graduation	76 (7.0)	37 (4.6)	39 (14.0)
University graduation	993 (91.8)	760 (94.5)	233 (83.8)
**Occupational status**	***p <* 0.0001** ^2^		
Unemployed or housewife	145 (13.4)	131 (16.3)	14 (0.5)
Student	423 (39.1)	346 (43.0)	77 (27.7)
Worker	71 (6.6)	40 (5.0)	31 (11.1)
Intermediate executive	132 (12.2)	84 (10.4)	48 (17.3)
Upper executive	272 (25.1)	186 (23.1)	86 (30.9)
Retiree	39 (3.6)	17 (2.1)	39 (7.9)
**Household size (*n* = 1059) ^3^**	***p =* 0.108** ^2^		
Living alone	29 (2.7)	17 (2.1)	12 (4.3)
Small family (≤4 members)	798 (73.8)	604 (75.1)	194 (69.8)
Large family (>4 members)	232 (21.4)	171 (21.3)	61 (21.9)
Unspecified	23 (2.1)	12 (1.5)	11 (4.0)
**Household welfare proxy (*n* = 1059) ^3^**	***p =* 0.14** ^2^		
Lower tertile	351 (32.5)	267 (33.2)	84 (30.2)
Middle tertile	353 (32.6)	251 (31.2)	102 (36.7)
Upper tertile	355 (32.8)	274 (34.0)	81 (29.1)
Not declared	23 (2.1)	12 (1.4)	11 (3.9)

^1^ Data are expressed as total number of respondents *n* with percentages between brackets (%). ^2^ *p-*value: null hypothesis of same distribution between both sexes (chi-squared and Fisher exact test); null hypothesis rejected at *p* < 0.05. ^3^ Number of respondents specifying their household size and welfare proxy.

**Table 2:** Anthropometrics and medical data of the participants.

	Total Population *n* (%) ^2^		Women *n* (%)		Men *n* (%)	
	1082 (100)		804 (74.3)		278 (25.7)	
Age (years) ^1^	32.5	12.0	31.1	10.8	36.5	14.2
Weight (kg) ^1^	71.1	15.2	67.8	13.9	80.5	14.8
Height (m) ^1^	1.68	0.08	1.64	0.06	1.77	0.06
BMI (kg/m^2^) ^1^	25.1	4.9	25.0	5.0	25.4	4.4
**Nutritional status ^2^**	***p* = 0.20 ^3^**					
Underweight	60 (5.5)		50 (6.2)		10 (3.6)	
Normal weight	528 (48.8)		396 (49.2)		132 (47.5)	
Preobese	337 (31.1)		241 (30.0)		96 (34.5)	
Obese	157 (14.5)		117 (14.6)		40 (14.4)	
**History of chronic diseases (number of diseases) ^2^**	***p* = 0.20** ^3^					
No chronic disease	852 (78.7)		632 (78.6)		220 (79.1)	
One disease	195 (18.0)		143 (17.8)		52 (18.7)	
Multiple diseases	35 (3.2)		29 (3.6)		6 (2.1)	
**History of chronic diseases (type of diseases) ^2^**	***p* = 0.002** ^3^					
Cardiovascular diseases	25 (10.9)		14 (8.1)		11 (19.0)	
Endocrine, nutritional, or metabolic diseases	56 (24.3)		37 (21.5)		19 (32.7)	
Respiratory and ENT diseases	14 (6.1)		8 (4.6)		6 (10.3)	
Digestive diseases	13 (5.7)		12 (7.0)		1 (1.7)	
Hematological diseases	26 (11.3)		26 (15.1)		0 (0.0)	
Genecology, urinary and kidney diseases	9 (3.9)		5 (2.9)		4 (6.8)	
Cancer	2 (0.9)		2 (1.2)		0 (0.0)	
Other diseases	50 (21.7)		39 (22.7)		11 (19.0)	
Morbid health status (more than 3 diseases)	35 (15.2)		29 (16.9)		6 (10.3)	
**Diet ^2^**	***p* = 0.004** ^3^					
Spontaneous feeding with no diet	960 (88.7)		713 (88.7)		247 (88.8)	
Healthy diet	23 (2.1)		15 (3.2)		8 (1.4)	
Weight-loss diet	34 (3.1)		29 (3.6)		5 (1.8)	
Reduced sugar/salt diet	20 (1.8)		8 (1.0)		12 (4.3)	
Therapeutic diet	13 (1.2)		11 (1.4)		2 (0.7)	
Vegetarian diet	2 (0.2)		2 (0.2)		0 (0.0)	
Not specified	30 (2.8)		26 (3.2)		4 (1.4)	
**Incidence of COVID-19** ^2^	***p* = 0.55** ^3^					
Not exposed	734 (67.8)		539 (67.0)		195 (70.1)	
Suspected infection	168 (15.5)		132 (16.4)		36 (12.9)	
Mid-form infection	167 (15.4)		124 (15.4)		43 (15.5)	
Severe form infection	13 (1.2)		9 (1.1)		4 (1.4)	
**Occurrence of family death due to COVID-19 ^2^**	***p* = 0.90** ^3^					
No	886 (81.9)		659 (82.0)		227 (81.6)	
Yes	196 (18.1)		145 (18.0)		51 (18.4)	
**Vaccination against COVID-19** ^2^	***p* = 0.14** ^3^					
No	847 (78.3)		638 (79.3)		209 (75.2)	
Yes	235 (21.7)		166 (20.7)		69 (24.8)	

^1^ Data are expressed as means with standard deviation in separate columns. ^2^ Data are expressed as total number of respondents with proportions between brackets for categorical variables. ^3^ *p*-value: null hypothesis of same distribution between both sexes (chi-squared and Fisher exact test); null hypothesis rejected at *p* < 0.05.

**Table 3:** Adherence to Mediterranean diet over sociodemographic data.

Item	MEDAS Score ^1^	Proportion of MD Adherents *n* (%) ^2^		
		Low Adherence	Medium Adherence	High Adherence
Total population (*n* = 1082)	6.6 ± 1.07 7 [6-8]	256 (23.7)	770 (71.2)	56 (5.2)
**Sex (*n* = 806) ^4^**	**Mann–Whitney test** ***p* = 0.24 ^3^**	**Chi-squared test** ***p* = 0.433 ^3^**		
Women	6.6 ± 1.7 7 [6-8]	145 (24.7)	409 (69.8)	32 (5.5)
Men	6.5 ± 1.6 6 [6-7]	50 (22.7)	162 (73.6)	8 (3.6)
**Location (*n* = 806) ^4^**	**Kruskal–Wallis test** ***p* = 0.028 ^3^**	**Chi-squared test** ***p* = 0.098 ^3^**		
Greater Tunis	6.5 ± 1.7 6 [5-8]	89 (25.2)	249 (70.5)	15 (4.2)
Northeast	6.5 ± 1.9 6 [5-8]	36 (28.8)	83 (66.4)	6 (4.8)
Northwest	6.41 ± 1.4 6 [6-7]	13 (19.4)	53 (79.1)	1 (1.5)
Center-east	7.1 ± 1.8 7 [6-8]	24 (17.5)	99 (72.3)	14 (10.2)
Center-west	6.3 ± 1.5 7 [5-7]	10 (27.8)	26 (72.2)	0 (0)
Southeast	6.6 ± 1.5 7 [5-8]	17 (25.7)	47 (71.2)	2 (3)
Southwest	6.5 ± 1.8 6 [5-8]	6 (27.3)	14 (63.6)	2 (9)
**Age category (*n* = 806) ^4^**	**Kruskal–Wallis test *p* < 0.001 ^3^** **Spearman test (r = 0.15, *p* < 0.001) ^3^**	**Chi-squared test** ***p* < 0.001 ^3^**		
Young adults (20–25 years)	6.3 ± 1.7 6 [5-7]	81 (30.6%)	176 (66.4)	8 (3)
Adults (25–60 years)	6.7 ± 1.7 7 [6-8]	113 (22.4%)	366 (72.5)	26 (5.1)
Elderly (over 60 years)	7.7 ± 1.8 7 [7-9]	1 (2.8%)	29 (80.5)	6 (16.6)
**Education** **(*n* = 806) ^4^**	**Kruskal–Wallis test** ***p* = 0.22 ^3^**	**Chi-squared test** ***p* = 0.768 ^3^**		
Not graduated	7.7 ± 0.9 7.5 [7-8.5]	0 (0)	4 (100)	0 (0)
Primary school graduation	7.5 [5.5-8]	2 (25)	6 (75)	0 (0)
Secondary school graduation	6.3 ± 1.4 6 [6-7]	12 (24.4)	36 (73.5)	1 (2)
University graduation	6.6 ± 1.7 7 [6-8]	181 (24.2)	525 (70.5)	39 (5.2)
**Occupational status** **(*n* = 806) ^4^**	**Kruskal–Wallis test** **(*p* = 0.015) ^3^** **Spearman test (r = 0.11, *p* = 0.017) ^3^**	**Chi-squared test** ***p* = 0.798 ^3^**		
Unemployed/housewife	6.5 ± 1.5 6.5 [5-8]	26 (26.5)	70 (71.4)	2 (2)
Student	6.4 ± 1.8 6 [5-8]	94 (29.3)	213 (66.3)	14 (4.3)
Worker	6.3 ± 1.3 6 [6-7]	14 (24.6)	43 (75.4)	0 (0)
Intermediate executive	6.4 ± 1.5 6 [6-7]	24 (24.7)	71 (73.1)	2 (2)
Upper executive	7 ± 1.8 [6-8]	36 (17.1)	154 (73.3)	20 (9.5)
Retiree	7.4 ± 1.5 7 [7-8]	1 (4.3)	20 (86.9)	2 (8.7)
**Household size (*n* = 788) ^5^**	**Kruskal–Wallis test** ***p* = 0.2 ^3^**	**Chi-squared test** ***p* = 0.079 ^3^**		
Living alone	6.3 ± 1.1 6 [6-7]	4 (21)	15 (79)	0 (0)
Small family (≤4 members)	6.7 ± 1.7 6 [6-8]	128 (21.6)	433 (73)	32 (5.4)
Large family (>4 members)	6.4 ± 1.8 6.5 [6-8]	55 (31.2)	32 (64.8)	7 (4)
**Household welfare proxy (*n* = 788) ^5^**	**Kruskal–Wallis test** ***p* < 0.001 ^3^** **Spearman test (r = 0.214, *p* <0.001) ^3^**	**Chi-squared test** ***p* < 0.001 ^3^**		
Lower tertile	6.17 ± 1.64 6 [5-7]	87 (33.1)	168 (63.9)	8 (3)
Middle tertile	6.55 ± 1.7 6 [6-8]	66 (23.6)	201 (71.8)	13 (4.6)
Upper tertile	7.11 ± 1.62 7 [6-8]	34 (13.9)	193 (78.8)	18 (7.3)

^1^ Data are expressed as means with standard deviation and medians with interquartile range between square brackets [IQR]. ^2^ Data are expressed as total number of respondents with proportions between brackets for categorical variables. ^3^ *p*-value: null hypothesis of same distribution between both sexes (chi-squared and Fisher exact test); null hypothesis rejected at *p* < 0.05. ^4^ Number of respondents under spontaneous feeding, healthy diet and sugar/salt-reduced diet. ^5^ Number of respondents specifying their household size and welfare proxy.

**Table 4:** Changes in smoking status and sleeping habits before and during the COVID-19 pandemic.

	Smoking Pre-COVID-19	Smoking during COVID-19
Nonsmoking	878 (81.2) ^1^	901 (83.2)
<5 cigarettes/day	49 (4.5)	37 (3.4)
5–10 cigarettes/day	44 (4.0)	48 (4.4)
>10 cigarettes/day	111 (10.2)	96 (8.8)
	**Sleeping habits pre-COVID-19**	**Sleeping habits during COVID-19**
<7 h/night	338 (31.2) ^1^	421 (38.9)
7–9 h/night	573 (53)	499 (46.1)
>9 h/night	171 (15.8)	162 (15)

^1^ Values are expressed as numbers and percentage (n (%)).

**Table 5:** Physical activities before and during COVID-19 pandemic.

	Sport Practiced Pre-COVID-19
None	505 (46.7) ^1^
Gym, yoga, dance, or aerobics	105 (9.7)
Walking	293 (27.1)
Swimming	7 (0.6)
Football, basketball, volleyball, or tennis	50 (4.6)
Martial arts	14 (1.3)
Two activities	95 (8.8)
Three activities or more	13 (1.2)
	**Sport practiced during COVID-19**
None	810 (74.9) ^1^
Gym, yoga, dance or aerobics	22 (2)
Weightless training	111(10)
Weight training at home	33(3)
Treadmill	16 (1.5)
Others	52(4.8)
Two activities	32(3)
Three activities or more	6 (0.5)

^1^ Values are expressed as numbers and percentage (n (%)).

## Data Availability

Not applicable.

## Appendix A. Questionnaire

**Table A1:** Sociodemographic and economic data.

Questions	Answers
1. How old are you?	Age in years
2. Sex	Man/woman
3. Nationality	Tunisian/other
4. Place of residence (governorate)	governorate
5. Instruction level	No schooling/primary schooling/secondary schooling/University graduate
6. Occupational status	Unemployed/housewife/student/worker/intermediate executive/upper executive/retiree
7. Number of people in the household	Number
8. What type of housing does the household have?	Housing type
9. Do you have electricity?	Yes/No
10. What is the source of drinking water for the family members?	Source of drinking water
11. Your status with regard to housing?	Owner/in ownership/renter/free accommodation with friends or relatives or company accommodation
12. How many rooms (for sleeping) do you have in your home?	Number of rooms
13. Do you have a refrigerator?	Yes/No
14. Do you have a washing machine?	Yes/No
15. Do you have a dishwasher?	Yes/No
16. Do you have a satellite dish?	Yes/No
17. Do you have internet access?	Yes/No
18. Do you have a television?	Yes/No
19. Do you have a central heating system?	Yes/No
20. Do you have an air conditioner?	Yes/No
21. Do you have a mobile phone?	Yes/No
22. Do you have a car?	Yes/No
23. Do you have a computer?	Yes/No

**Table A2:** Anthropometrics and medical data.

Questions	Answers
1. Enter your weight (kg) as accurately as possible.	Weight in kg
2. Enter your height (cm) as accurately as possible.	Height in cm
3. Do you suffer from any chronic illness? If yes, quote, otherwise write No.	Yes/No
4. Do you follow a special diet? If yes, specify, otherwise write No.	Yes/No
5. Did you contract COVID-19?	No/Yes, tested positive with mild form of the disease/Yes, tested positive with severe form of the disease/suspected positive/contact case
6. Have you experienced family death due to COVID-19?	Yes/No
7. Have you had a vaccine against COVID-19?	Yes/No

**Table A3:** MD adherence.

Questions	Answers
1. Is olive oil the main culinary fat used?	Yes/No
2. How many teaspoons of olive oil do you consume per day (including that used for frying, salads, eating out, etc.)?	4 or more/2 or 3/1 or less
3. How many portions of vegetables (cooked and raw potatoes and beans are not included) do you eat per day? (One portion = half a large plate)	3 or more/1 or 2/less than 1
4. How many servings of fresh fruit do you consume per day? (Serving size = one unit of medium-sized fruit, one large cup of sliced fruit, one slice of medium-sized melon/watermelon, or one cup of fresh juice)	3 or more/1 or 2/less than 1
5. How many servings of red meat (veal, beef, mutton)/hamburgers/other meat products (salami, merguez, sausage…) do you consume per day? (One serving = 100 to 150 g = one quarter to half of a meal dish)	1 or less/2 to 4/5 to 6/7 or more
6. How many portions of butter, margarine or cream do you consume per day? (One portion = 12 g = one dessert spoon for butter and margarine, 2 tablespoons for cream)	Less than 1/one/more than 1
7. How much sugary drinks (industrial juices)/carbonated drinks/sodas do you consume per day? (One serving equals 330 mL)	Less than 1/1 or more
8. How many glasses/cups of wine do you consume per week?	More than 14 glasses (more than 2 glasses per day)/7 to 14 glasses (1 or 2 glasses per day)/2 to 6 glasses (sometimes but not every day)/one glass or less (occasionally)/none
9. How many portions of legumes (lentils, beans, chickpeas, peas…) do you consume per week? (One portion = 150 g = one dish or glass)	3 or more/1 to 2/less than 1
10. How many portions of fish and seafood do you eat per week? (One portion = 100 to 150 g = a quarter to half of a meal dish)	3 or more/1 to 2/less than 1
11. How many times a week do you consume industrial (not homemade) desserts/confectionery/pastries? (Including cakes, biscuits, ice cream, etc.)	Less than 1/1 to 2/3 or more
12. How many servings of nuts (unsalted) do you consume per week? (Including unsalted peanuts, almonds, hazelnuts, walnuts, etc. One serving = 30 g = one handful)	3 or more/1 to 2/less than 1
13. Do you prefer to consume chicken, turkey, or rabbit meat, or a vegetarian protein source, rather than red meat or derived products?	Yes/No
14. How many times a week do you eat dishes cooked with tomatoes or tomato sauce, onion and/or garlic and olive oil?	Twice or more/once or less

**Table A4:** Eating habits related to Mediterranean diet.

Questions	Answers
1. How many portions of pasta, rice, or other cereals including Oriental and Western pastries do you consume per day?	None/half portion/1 portion/2 portions/more than 2 portions
2. How many servings of whole meal, cracked wheat, cooked wheat, and corn bread do you eat per day? (1 average serving = 80 g or 2 slices)	None/half portion/1 portion/2 portions/more than 2 portions
3. How many servings of milk or yoghurt do you consume per day (1 serving = 150 mL in a cup or 125 g in a pot)?	None/half portion/1 portion/2 portions/more than 2 portions
4. How many servings of cheese or dairy products do you consume per week (1 serving of dairy product = 100 g; 1 serving of ripened cheese = 50 g)?	None/half portion/1 portion/2 portions/more than 2 portions
5. How many eggs do you consume per week?	None/1 egg/2 eggs/4 eggs/more than 4 eggs
6. How much water did you drink per day during the third wave of the pandemic?	Less than 1 L/1 to 2 L/more than 2 L

**Table A5:** Lifestyle habit changes.

Questions	Answers
1. Did you smoke before COVID-19 (cigarettes, cigars, electronic cigarettes)?	No/Yes, fewer than 5 cigarettes/Yes, between 5 and 10 cigarettes/Yes, more than 10 cigarettes
2. Do you currently smoke?	No/Yes, fewer than 5 cigarettes/Yes, between 5 and 10 cigarettes/Yes, more than 10 cigarettes
3. Your sleeping habits before COVID-19?	Less than 7 h per night/7 h to 9 h per night/More than 9 h per night
4. Your sleeping habits at present?	Less than 7 h per night/7 h to 9 h per night/More than 9 h per night
5. Did you play any sport before COVID-19?	No/gymnastics, yoga, dance, aerobics/walking/swimming/football, basketball, volley, tennis/martial arts
6. Do you currently play sports at home?	No/Yes, training without weights/Yes, weight training at home/yoga/treadmill/postural gymnastics/others
7. How many times did you play sports during COVID-19?	I didn’t play sports/1 to 2 times per week/3 to 4 times per week/more than 4 times per week
